# Women’s perception of continuity of team midwifery care in Iran: a qualitative content analysis

**DOI:** 10.1186/s12884-021-03666-z

**Published:** 2021-03-02

**Authors:** Sholeh Shahinfar, Parvin Abedi, Mahin Najafian, Zahra Abbaspoor, Eesa Mohammadi, Narges Alianmoghaddam

**Affiliations:** 1 Department of Midwifery, Nursing and Midwifery School, Ahvaz Jundishapur University of Medical Sciences, Ahvaz, Iran; 2 Department of Midwifery, Reproductive Health Promotion Research Center, Ahvaz Jundishapur University of Medical Sciences, Ahvaz, Iran; 3 Department of Obstetrics and Gynecology, School of Medicine, Fertility Infertility and Perinatology Research Center, Ahvaz Jundishapur University of Medical Sciences, Ahvaz, Iran; 4 Department of Midwifery, Reproductive Health Promotion Research Center, Ahvaz Jundishapur University of Medical Sciences, Ahvaz, Iran; 5 Department of Nursing, Faculty of Medical Sciences, Tarbiat Modares University, Tehran, Iran; 6 School of Public Health, Massey University, Palmerston North, New Zealand

**Keywords:** Perception, Continuity of care, Qualitative research, Empowerment

## Abstract

**Background:**

Understanding the pregnant women’s perception of continuity of team midwifery care is necessary for introducing and implementing this model of midwife-led care in the Iranian maternity services. This qualitative study aims to explore women’s perception of continuity of team midwifery care in Iran.

**Methods:**

This research is a qualitative study conducted in Iran to explore women’s perception of continuity of team midwifery care during pregnancy, birth and postpartum from October 2019 to August 2020. Fifteen semi-structured interviews were conducted with women individually in private midwifery clinic through a purposive sampling method. Interviews were digitally recorded and transcribed verbatim in Persian and analyzed using conventional content analysis.

**Results:**

From the data analysis, two themes, four main categories, and nine subcategories emerged. The themes were “Maternal empowerment” and “Mother’s satisfaction during the transition from pregnancy to motherhood”. The first theme included two categories of improving self-efficacy during antenatal education classes and the effective midwife-mother interaction. The second theme composed of two categories of satisfaction with the process of pregnancy, childbirth and postpartum as well as satisfaction with motherhood.

**Conclusion:**

Findings of this qualitative study highlight the effectiveness of continuity of team midwifery model of care for promoting empowerment and satisfaction in women during pregnancy, birth and postpartum. The results of this study could pave the way for developing, introducing and implementing the midwife-led continuity models of care in Iran.

**Supplementary Information:**

The online version contains supplementary material available at 10.1186/s12884-021-03666-z.

## Background

The experiences of pregnancy, giving birth and having a baby create lifelong memories for women. The impact of maternity experiences on women can be empowering and lead to feelings of self-worth and self-confidence or may cause negative maternal health outcomes such as postpartum depression and post-traumatic stress disorder [1, 2]. The quality of maternity services is the main aspect of these experiences and midwives have a pivotal role in providing high-quality services to promote a positive birth experience for women and their family [3–5].

Research has shown that maternity service standards in Iran, need to be reviewed effectively to implement a high-quality and safe women-centred maternity care service for women and their family [6]. Although about 90% of Iranian women choose hospital to give birth and statistics have shown a 75% reduction in the maternal deaths up to 2015, unnecessary medical interventions still are very common during the physiologic normal labour and birth in Iran compared to developed countries [7, 8]. Medical dominance and the fragmented maternity care in Iran do not pay attention to the significance of women-midwife relationship during pregnancy, birth and postpartum [5, 9]. Furthermore, some sociocultural factors cause the maternity care problems in Iran. For example in an ethnographic study in Iran, it was found that cultural norms, values and social network can lead to increased cases of cesarean delivery [10]. Hence, finding new paths to address these problems while maintaining high maternity service standards is an important goal in many countries.

During four decades, midwives have been able to establish private clinics and visit pregnant women as part of their job responsibilities privately [11]. Some of them provide midwifery continuity of care throughout the antenatal, intrapartum and postpartum periods, but in some midwifery clinics, the care is limited to antenatal and/or postpartum periods. During recent decades, labour/birth care is usually provided by midwives in private hospitals. Totally, maternity care in Iran is obstetrician-led and though the midwife might be the main care provider, but the obstetrician undertakes all the responsibilities of caring for the woman from the antenatal to the postpartum continuum [12]. There is not team midwifery model of care in Iran.

It has been proven that continuity of midwifery care is the best model of maternity care for mothers and babies, and this model of care is growing in countries with high standards of living such as New Zealand, Australia, the United Kingdom, and Denmark [13]. The continuity of midwifery of care is a model of care provided by a known caseload midwife or a team of known caseload midwives from early pregnancy until 6 weeks after birth [14]. This model of care is a process through which midwives provide holistic care and recognize woman’s social, emotional, physical, spiritual and cultural needs [15]. Moreover, evidence-based research has shown that continuity models of midwifery care lead to a reduced rate of unnecessary medical interventions as well as improved maternal and neonatal health outcomes [12, 16]. Also, such models of care have a positive impact on mothers’ satisfaction and appear to be cost-effective for improving maternal health services [17–19]. Fawsitt et al. in a qualitative study of continuity of midwifery care found that women appreciated the continuity of care which was received by the same midwives throughout their pregnancy and birth because it provided a sense of safety [20]. Hildingsson et al. also reported that women who received care from a known midwife were more likely to have a positive birth experience in terms of professional support [13].

The advantages of continuity of midwifery care for improving maternity services is evident. However, regarding medical dominance in the maternity system, more efforts are needed to assess such midwife-led models as the novel models of maternity care in Iran. Since the availability, efficacy and acceptability of different models of care depend on the local context, we performed a large project to check the effect of team midwifery care on the outcome of pregnancy and also a qualitative study on the perception of women about team midwifery care [21]. In this manuscript we will report the results of qualitative study.

## Methods

### Study design and setting

The present article is part of a larger mixed-method study that aimed to evaluate the effect of continuity of team midwifery care on pregnancy outcomes in the city of Ahvaz, Iran. The paradigm of this research is pragmatism, which is associated with mixed methods and works most effectively for the research problem [22]. A qualitative approach has been chosen as a complement to quantitative assessments because fixed clinical measures are inadequate to explain the complexity of human experiences, particularly childbirth [23]. Among various types of qualitative research methods, content analysis has been used to “provide knowledge and understanding of the phenomenon under study” and explore the mothers’ perception of continuity of team midwifery care during pregnancy, birth and postpartum from October 2019 to August 2020 [24]. Women who had rich experiences of the pregnancy, birth and postpartum periods and also ability to express them, were selected (purposive sampling). Although the women who came to the private midwifery clinic were somewhat similar in some characteristics, such as cultural and economic status, it was tried to have the maximum variety in sampling.

The study settings were a private midwifery clinic and two hospitals (Sina Hospital and Alameh Karami Hospital) in Ahvaz city, Iran. The main reason for choosing these hospitals as study settings was that the two research assistants (midwives) deliver babies in there. The first hospital is a state hospital and the latter is a charitable public hospital which they manage about 3500 and 3000 births per year respectively. Midwifery continuity of care is not offered in these hospitals like other hospitals in Iran, because the prenatal care is provided by other midwives employed in primary health center or private midwifery clinic. Midwives in mentioned hospitals, provide the standard care and work under the supervision of obstetricians.

The private midwifery clinic was selected for providing team midwifery model of care. A team of three licensed midwives including two self-employed midwives and one doctoral student in midwifery (SS), provided continuity of care for the research participants throughout pregnancy, birth and the postpartum period. Women’s booking visit with the midwifery team was earlier than 24 weeks of pregnancy. Prenatal care was provided in the private midwifery clinic. The midwives presented in the private midwifery clinic every other day, but the team midwives were 24 h/7d on call. All women gave birth in those aforementioned hospitals assisted by one of the team midwives who was on call at that time and minimum two routine postpartum visits were performed for the mother and babies during the first 6 weeks after birth. The midwifery care plan was in accordance with the national maternity guideline and in case of occurring any complication, participants were referred to an obstetrician for consultation [25]. Also, all women gave birth in those mentioned hospitals assisted by one of the team midwives who was on call at that time and minimum two routine postpartum visits were performed for the mother and babies during the first 6 weeks after birth. The participants of the present research were the eligible women who completed the quantitative part of the study.

### Ethical considerations

Ethical approval for this study was granted by the Ethics Committee of Ahvaz Jundishapur University of Medical Sciences (IR.AJUMS.REC.1398.096). Confidentiality of the collected data was ensured, and written informed consent was obtained from all research participants. Also, participants were free to decline participation or withdraw at any stage of the research process. All interviews were recorded with the permission of participants and all the audio files were securely stored in the password-protected computers.

### Data collection and participants

In-depth, semi-structured interviews were conducted, digitally recorded and transcribed into the original language (Persian). Interview were recorded using a Sony ICD BX 470 recorder. Data were collected individually via face to face or telephone interviews by the first author. The interviews were carried out in a separate room at private midwifery practice and no one other than the interviewer and participant was present in the room. Due to COVID-19 pandemic and the lockdown in Ahvaz city some of the interviews conducted by phone. During this period of time, when pregnant women encountered a complication, they called team midwives and consulted with them. Women were called by prior arrangement and interviews were recorded by mobile phone call recorder. An interview schedule containing three open-ended questions was used. Participants were asked to explain their perception of care received from the midwifery team during pregnancy, birth and postpartum. The first question was “Would you describe your perception of care received from team midwives during pregnancy?” And then participants were asked about the quality of care they had received during their childbirth and postpartum. Probing questions were asked when more clarifications were needed. The duration of each interview varied from 45 to 60 min. All interviews continued until data saturation reached and no new concept was added. Fifteen primipara women who met the inclusion criteria participated in this research voluntarily. Inclusion criteria were as follows: nulliparity, term singleton delivery, low-risk women who are eligible for receiving maternity care from a midwife, age 18 years or older, ability to provide sufficient information on the subject and willingness to participate in research and sign the informed consent form.

### Data analysis

Data analysis performed using conventional content analysis method, guided by Graneheim and Ludman in five stages [26]. Also, concurrent data collection and analysis were used. Firstly, the interviews were digitally recorded and transcribed verbatim. Secondly, each interview transcript was read and re-read several times to identify meaning units. Thirdly, the condensed meaning units were abstracted and labelled with codes. Then the codes were classified into subcategories and then categories derived based on comparisons regarding conceptual and semantic similarities. Finally, after comparing categories and subcategories together, themes were extracted from the analysis and interpretation of the qualitative data. Data analysis process was performed using the MAXQDA software (version 10).

All the interviews were performed and coded initially by the first author, who was fluent in original language (SS). To ensure the credibility of research data, sampling was done until data saturation, appropriate meaning units were selected and participants’ checking was carried out. To strengthen conformability, a detailed description of the research steps was presented to and then confirmed by all co-authors. Dependability was ensured by the authors describing the analysis process. Transferability of the findings was gained as the results were logical for three women who experienced team-midwifery care but did not participate in this study.

## Results

The findings emerged from the data analysis of the face to face and phone interviews with fifteen primipara women who participated in this research. The average age of the participants was 24 years old. Table 1, represents the demographic characteristics of the research participants. From the data analysis, two themes, four main categories, and nine subcategories emerged (Table 2). The themes were “Maternal empowerment” and “Women’s satisfaction during the transition from pregnancy to motherhood”.

**Table 1:** Demographic Characteristics of participants (*N* = 15)

Variable	N (%)
**Age group**	
18–25	9 (60)
26–35	6 (40)
**Education level**	
High school	6 (40)
University	9 (60)
**Employment status**	
Housewife	11 (73.33)
Employee	4 (26.67)
**Abortion**	
0	13 (86.66)
1	2 (13.34)
**Gravidity**	
1	13 (86.66)
2	2 (13.34)

**Table 2:** Categories, subcategories and Themes

Categories and subcategories	Themes
***Improving self-efficacy during antenatal education classes*** Increased knowledge and skills of couples The mother’s ability to cope with new situations Emotional acceptance of natural birth by mother	***Maternal empowerment***
***The effective midwife-mother interaction*** Mother’s trust in the midwife Mother’s perceived support from the midwife	
***Satisfaction with the process of pregnancy, childbirth and postpartum*** Satisfaction with pregnancy period Satisfaction with childbirth and postpartum periods	***Mother’s satisfaction during the transition from pregnancy to motherhood***
***Satisfaction with motherhood*** A sense of pride through passing the difficult path of childbirth More motivation for acceptance of getting pregnant again	

### Maternal empowerment

Empowerment is a woman’s ability to take the lead in the decision making process about her maternity care through interaction and information sharing with her chosen midwife [27]. This theme consisted of two categories as follows: Improving self-efficacy during antenatal education classes and the effective midwife-mother interaction.

### Improving self-efficacy during antenatal education classes

Self-efficacy is women’s belief in their ability to perform a task or behaviour [28]. In this current study, participants expressed improvement in their self-efficacy through antenatal education classes which grouped in three subcategories of increased knowledge and skills of couples; the mother’s ability to cope with new situations; and emotional acceptance of physiological birth by mother.

#### Increased knowledge and skills of couples

Participants frequently mentioned that they were satisfied with the antenatal classes and explained that they did not have any prior knowledge regarding pregnancy and childbirth as a first-time mother. They asserted that these classes improved their knowledge and skills, and then they transferred this knowledge to their partners.

*“I was very inexperienced; I didn’t know anything at all. As soon as I came here (antenatal classes), they informed me about the physiological pregnancy and birth process and what may happen”* (Woman 5)

*“When I came for antenatal classes around 32-34 weeks of my pregnancy, I’m talking about the pain relief classes, you taught me about several pressure points … In case of I may forget those things during the labour and birth, I had written and gave it to my husband. When the pain started, he massaged those points. Especially the one which was on my foot, helped me a lot”* (Woman 6)

#### The mother’s ability to cope with new situations

From the participants’ point of view, antenatal classes empowered them to cope with the new challenges that they faced during the physiological process of pregnancy, birth and postpartum. Additionally, they learned how to manage and deal with labour pain, according to their individual needs and situations.

***“****I didn't expect that childbirth is a very simple task, but with the educational classes and what you taught us, such as exercises, the process became much easier.”* (Woman 5)

*“I didn’t know what to do, whenever I had pain. For example, sometimes I had a stomachache, I didn’t know where to go, I was thinking maybe I should go to hospital. But after participating in these antenatal classes, everything was explained to us. I was doing the same thing that you told us and my pain was really reduced.”*(Woman 10)

*“I was told in those classes … this (labour pain) pain comes and goes. The moment you are pain-free and between pains and you are Okay ...mobilise and do the things you like. It helped me a lot. I mean, I was at home at 4.30 am, and I had severe pain. When the pain wasn’t there, for example, I was walking, filling the hot water bottle, packing my stuff for the hospital stay (getting ready to go to the hospital) and then, when the pain started, I had to sit again. It was so good.”*(Woman 8)

#### Emotional acceptance of natural birth by mother

The majority of women who participated in this research reported that antenatal classes played a remarkable role in reducing their fear and anxiety. Moreover, these classes helped them to enhance their pain tolerance, change their negative attitude towards birth and demystify their misconceptions about childbirth.

*“When I was with my midwife ... Her help, her words were very soothing. She guided me before my pain was started. I was fearful, I was very anxious, but she helped me a lot.”* (Woman 4)

*“I wasn’t tolerant. I couldn’t tolerate this pain at all, could I? Not at all! To bear the pain of having a baby. But I became more patient when I came to these classes.”* (Woman 2)

*“When I attended those classes, they (the team midwives) said … the body changes naturally. It changes during natural birth … When I got this information, my mind got ready and accepted it. So, such a thing is possible. When my mind accepts such a thing, the body naturally accepts it”* (Woman 7)

### The effective midwife-mother interaction

This category brings together two subcategories that emerged from the data: Mother’s trust in the midwife and mother’s perceived support from the midwife.

#### Mother’s trust in the midwife

Participants said that they followed the advice given by the midwives because they believed that the team midwives were very knowledgable, skilled and experienced as well as very compassionate midwives who they could trust them.

*“Yea! In one of my blood tests, my haemoglobin was a bit low. She (the midwife) gave me some advice. I did the same. My vitamin D also was low. But the next blood test came back normal. It was good. I could trust her ... trust. She (the midwife) is very experienced, she is very sympathetic.” (*Woman *5)*

Some participants claimed that the partnership relationship with the midwife was one of the reasons which they felt relaxed and could express their problems and ask their questions. They considered the team midwives as their professional friends who provided women-centred continuity midwifery care for them:

*“If I want to get pregnant again, I will choose her again (one of the team midwives). She is very friendly and patient. This relationship has a great effect on you to be comfortable with her. She is like your mother, your sister ... you are not afraid of having a problem. You would tell her.”* (Woman 1)

#### Mother’s perceived support from the midwife

All research participants mentioned that the team midwives were very approachable, accessible and they were always available for them which gave them a feeling of assurance.

***“ … …*** *Her words, her help, were very effective, for example when I was in pain or I had a question, even in the middle of the night, I sent her a text and she replied immediately. It reassured me that I knew I had a midwife who is always there for me, she could help me, she could answer my questions very well, she could take care of me.”* (Woman 4)

Participants appreciated companionship and support of midwives during the birth process, which led them to feel empowered and safe:

*“When I realized that I’m in labour, I was happy. My mum said: “aren't you scared, love?” I said “no, mom”, I’m happy. The reason that I was happy was the guidance of Mrs. ... (midwife), her support. That I knew someone is with me. Nothing terrible will happen because I’m in safe hands.”* (Woman 8)

### Mother’s satisfaction during the transition from pregnancy to motherhood

This theme included two categories of satisfaction with the process of pregnancy, childbirth and postpartum as well as satisfaction with motherhood.

### Satisfaction with the process of pregnancy, childbirth and postpartum

This category involves two subcategories of participants’ satisfaction with pregnancy period and participants’ satisfaction with childbirth and postpartum periods.

#### Satisfaction with pregnancy period

This subcategory represents the positive viewpoint of participants about avoiding unnecessary interventions and relying more on experience and knowledge in the process of care by midwives. Some participants appreciated midwives’ efforts to encourage them giving birth normally and avoid caesarian section which is not medically indicated.

*“On the other hand, I didn’t want to go to ultrasound all the time. They (midwives) could understand a series of issues with an examination and according to their experience. They could understand even the rotation of the baby with the examination. Well, this was much better for me.* “(Woman 11)

*“I even think, I bothered her (midwife) a lot. For example, at the end of the labour, I said: “call my husband and let him sign for me to have a cesarean section.” Then, she said: "No, I’m not a midwife who let you undergo the surgical blade, I don’t allow that, at all.”* (Woman 12)

Based on participants’ statements, in addition to midwives’ clinical skills, their accompaniment during pregnancy enhanced their inner strength and made this period memorable for them.

*“I nearly experienced a very good year. I mean, nine months. It takes about a year for my baby to be born and somehow grow. My tolerance was enhanced a lot when I was seeing her (midwife) accompanied me (during labour) patiently.”* (Woman 4)

### Satisfaction with childbirth and postpartum periods

All participants were grateful with giving birth by a known midwife, as they were relaxed and was not anxious. They stated that following a pleasurable physiological birth, they got a new and positive attitude towards labour and recommended it to others because they believed in their care providers and their abilities.

*“They (midwives) give birth themselves. Women usually do like to be examined by someone every minute when they go to the hospital. I didn’t want someone to come every minute, for example, I was embarrassed. They (team midwives) only examined me themselves. It was very good, I was satisfied.”* (Woman14)

*“I told them, the ladies … (midwives) are angels. With their help, I had no pain at all. You would have neither pain nor stress. Having these people, it is very easy to give birth.”* (Woman 12)

Participants were satisfied with their midwives because they were so promising and helped them to relieve their concerns during the postpartum period:

*“At early pregnancy, I wasn’t worried. But in the end, it was very difficult. Both birth and becoming a mother made me concerned. It is a tough responsibility. I was worried about my studies, my job … that I couldn’t take care of my baby. Most of these issues were my concerns. Mrs ... (midwife) helped me a lot.”* (Woman 11)

### Satisfaction with motherhood

This category comprises of two subcategories of a sense of pride through passing the difficult path of childbirth and more motivation for acceptance of getting pregnant again.

**A sense of pride through passing the difficult path of childbirth** women were pleased with their experience of natural labor, which offered them a great sense of triumph and dignity. Some of them enjoyed having a beautiful feeling of accomplishment, self-confidence and empowerment following giving birth

*“... (With the birth of the baby) as if putting the world on your chest. Something very big happened, that you could handle. Yes, I always say it is a miracle. Why do they say now it is not a miracle? ... Every time a baby is born, a miracle happens. “*(Woman 1)

*“When my baby was born, the moment of his birth was very beautiful. Something that has not experienced before. One feels that has been born again. A sense of pride and confidence. You are complete. This feeling was so beautiful.”* (Woman 4)

*“(Birth) made me very strong … My self-confidence improved, when I could bear it all night. Birth is a difficult path for every woman.”*(Woman 7)

#### More motivation for acceptance of getting pregnant again

According to statements of some participants, they did not afraid of getting pregnant again, due to experience of a comfortable birth and even one of the women pointed out that following her natural birth, she decided to get pregnant again, because of her amazing midwife.

*“Two months ago, I thought I was pregnant. I mean, I had this stress. But I wasn’t afraid. I said that if I was probably pregnant and wanted to give birth, I would have no problem. I mean, I wasn’t afraid of pregnancy and childbirth. This is because I had a comfortable birth.”* (Woman 9)

*“When I got pregnant, I told my husband “That’s it, I’m done”. After I gave birth, I told him that I want another girl. He asked me “Do you want to get pregnant again?” I told him that yes I do, because of having Mrs ... (midwife), I want to get pregnant again, I want another girl.”* (Woman 2)

## Discussion

This qualitative research aimed to gain a deeper understanding of women’s perception of continuity of team midwifery care during pregnancy, childbirth and postpartum in Iran. The women’s perceptions were categorized into two themes: “Maternal empowerment” and “Mother’s satisfaction during the transition from pregnancy to motherhood”.

According to the obtained results, antenatal classes improved women’s self-efficiency, which was one of the significant findings of this study as the research. This result aligns with Byrne et al. (2014), who concluded that participants in childbirth education programs had significantly more self-efficacy and positive expectations of their birth [29].

Women in this study regarded self-efficacy as enhanced knowledge and coping ability during pregnancy as well as emotional acceptance of natural birth. Research has shown that childbirth is a broad range of uncertainties especially for new mothers [30]. Participants claimed that they were inexperienced and unaware of pregnancy and birth as first-time mothers and antenatal classes by the team midwives, improved their knowledge and skills during pregnancy. The information acquuired by women throughout pregnancy, give them enough knowledge to be involved in decision making and achieve a feeling of control and safety [31]. In our research, participants refered to their increased ability to cope with unexpected situations during their pregnancy and childbirth as one of the positive aspects of the antenatal classes. They learned how to handle their psychological and anatomical changes during pregnancy, and how to respond to these deviations. Hence the journey of pregnancy was facilitated for them and got more prepared to encounter with its difficult parts [32]. In addition, Participants pointed out that they got emotionally prepared for accepting natural birth. However, quality strategies included antenatal education are important for helping women to get prepared for labor and birth, but the midwives’ communication skills are essential in this regard, which had a main role in *women’s satisfaction* with team midwifery model of *care in our study* [31].

Moreover, in the present research, women reported the effective midwife-mother interaction in team-midwifery care as they could trust the midwives. Participants noted that they follow the advice that was given by the team midwives due to their skills, knowledge and expertise in midwifery. Our findings are in accordance with the study conducted by Perriman et al.(2018) who indicated that when events did not go as planned, women were following the recommendation that was given by their midwife [27]. Being compassionate and empathetic alongside having midwifery knowledge and competencies were significant characteristics of the midwives which led to developing trust in the women [31]. Participants appreciated midwives because of providing a friendly and trustful atmosphere to talk and ask questions. If the midwife is a good communicator, the woman can ask questions and understand the answers and she feels respected and valued [32]. Trusting relationship between women and midwives is a key determinant of respectful maternity care [33].

The participants in this study considered the effective midwife-mother interaction in team midwifery care as the mother’s perceived support from the midwife. They were satisfied with the easy and constant access to midwives, which is important for developing a trusting relationship between them and enhancing the probability of having good birth outcomes [31]. Women appreciated the emotional support and presence of midwives during labour which empowered them to accomplish the process of labour and birth [34].

We found that women were satisfied with the team-midwifery care during pregnancy and their reliance on clinical examination and experience instead of unnecessary medical interventions. On the contrary, Hunter et al. (2017) in their thematic analysis of women’s and clinicians’ experiences reported that women regarded medicalized care as the same as normality because of its safety [35]. While Miller et al. (2016) noted that inappropriate or routine use of interventions would be harmful and women are frequently not informed of the risks, which is similar to our findings [36].

The current research participants also were satisfied with team-midwifery care during labour and postpartum. Women valued giving birth with a known midwife. One of the reasons was that they got embarrassed to be examined by different care providers, which is in line with the Hassan et al.’s (2012) study [37]. Some women wanted to share the inspiring and pleasant experience of natural birth and recommended it to future mothers. Women were grateful of midwives who helped them to deal with the difficulties of their postpartum period that supports the Lyberg and Severinsson’s (2010) study result [38]. If women have a place to rely on after their discharge, they would have the confidence to overcome difficulties, because of their sense of security [39].

Participants considered Satisfaction with motherhood as a sense of self-confidence, great success, pride, empowerment and even happening miracle. One of the mothers expressed her feeling of being able to manage labour pains as an accomplishment, which is one aspect of positive birth experience [40]. These significant experiences are aligned with other studies [41, 42].

Redshaw et al.(2019) suggested that a woman’s experiences of maternity care might have been linked to her future pregnancies and also decision-making about future childbirth [1]. In this study, several women stated that they did not afraid of getting pregnant again and one of them had a tendency towards second pregnancy due to the quality care received from team midwives. However, in addition to the quality care, confidence which is related to sympathetic attitude towards childbearing could lead to positive expectations towards the coming birth [41].

We are aware that our study may have two limitations. One of the limitations is related to the (*COVID*-19) Pandemic and the lockdown in the middle of the collecting data, which we had to interview with nearly half of the participants by telephone. Hence, in addition to the lack of face-to-face communication, their perception of care might have been affected by the pandemic situation. Also, sampling in a private midwifery office was the second limitation of the present study, as we could not have the optimal maximum variety in sampling.

## Conclusion

Understanding the women’s perception of continuity of team midwifery care is necessary for developing, introducing and implementing this model of midwife-led care in Iran. Therefore, this qualitative study conducted to highlight the significance of women-midwife relationship during pregnancy, birth and postpartum which is ignored by the dominant fragmented maternity care system. Findings of this study emphasize the effectiveness of continuity of team midwifery model of care for promoting empowerment and satisfaction in women during pregnancy, birth and postpartum.

### Acknowledgements

The authors would like to thank all the women who participated in this study.

## Supplementary Information

**Additional file 1.**

## Data Availability

All relevant data are given within the manuscript and the supplementary files.

## Abbreviations

COVID-19: Coronavirus disease of 2019
COREQ: Consolidated criteria for reporting qualitative research

## References

[1] Redshaw M, Martin CR, Savage-McGlynn E, Harrison S (2019). Women’s experiences of maternity care in England: preliminary development of a standard measure. BMC Pregnancy Childbirth..

[2] Carquillat P, Vendittelli F, Perneger T, Guittier M-J (2017). Development of a questionnaire for assessing the childbirth experience (QACE). BMC Pregnancy Childbirth..

[3] Renfrew MJ, McFadden A, Bastos MH, Campbell J, Channon AA, Cheung NF (2014). Midwifery and quality care: findings from a new evidence-informed framework for maternal and newborn care. Lancet.

[4] Dahlberg U, Persen J, Skogås A-K, Selboe S-T, Torvik HM, Aune I (2016). How can midwives promote a normal birth and a positive birth experience? The experience of first-time Norwegian mothers. Sex Reprod Healthcare.

[5] Dahlberg U, Aune I (2013). The woman's birth experience—the effect of interpersonal relationships and continuity of care. Midwifery..

[6] Firouznia R, Dargahi H, Jafari Koshki T, Khaledian Z. Challenges of Iranian Maternal Health Program from Midwives’ Perspectives: A Qualitative Study. Jundishapur J Health Sci. 2019;11(3):e92354. 10.5812/jjhs.92354.

[7] Faghani Aghoozi M, Amerian M, Mohammadi S, Yazdanpanah A, Azarabadi S (2020). A review of the quality of midwifery care in Iran. Educ Ethic Nurs.

[8] Firooznia R, Dargahi H, Jafari-Koshki T, Khaledian Z (2018). Strategic analysis of maternal health program evaluation system for providing improvement strategies using internal-external environment assessment technique in Iran. Manage Strateg Health Syst.

[9] Aune I, Dahlberg U, Ingebrigtsen O (2011). Relational continuity as a model of care in practical midwifery studies. Br J Midwifery.

[10] Roudsari RL, Zakerihamidi M, Khoei EM (2015). Socio-cultural beliefs, values and traditions regarding women’s preferred mode of birth in the north of Iran. Int J Community Based Nurs Midwifery.

[11] Hakimi S. A century (1919-2019) of academic midwifery in Iran: from traditional midwives to PhD graduates. Eur J Midwifery. 2019;3(June):11. 10.18332/ejm/110065. 10.18332/ejm/110065 PMC7839103 33537590

[12] Sandall J, Soltani H, Gates S, Shennan A, Devane D. Midwife-led continuity models versus other models of care for childbearing women. Cochrane Database Syst Rev. 2016;4:CD004667. 10.1002/14651858.CD004667.pub5. 10.1002/14651858.CD004667.pub5 PMC8663203 27121907

[13] Hildingsson I, Karlström A, Larsson B. Childbirth experience in women participating in a continuity of midwifery care project. Women Birth. 2020. 10.1016/j.wombi.2020.04.010. 10.1016/j.wombi.2020.04.010 32595033

[14] Homer CS (2016). Models of maternity care: evidence for midwifery continuity of care. Med J Aust.

[15] Yanti Y, Claramita M, Emilia O, Hakimi M (2015). Students’ understanding of “women-Centred care philosophy” in midwifery care through continuity of care (CoC) learning model: a quasi-experimental study. BMC Nurs.

[16] Gidaszewski B, Khajehei M, Gibbs E, Chua SC (2019). Comparison of the effect of caseload midwifery program and standard midwifery-led care on primiparous birth outcomes: a retrospective cohort matching study. Midwifery..

[17] Tracy SK, Welsh A, Hall B, Hartz D, Lainchbury A, Bisits A (2014). Caseload midwifery compared to standard or private obstetric care for first time mothers in a public teaching hospital in Australia: a cross sectional study of cost and birth outcomes. BMC Pregnancy Childbirth.

[18] Forster DA, McLachlan HL, Davey M-A, Biro MA, Farrell T, Gold L (2016). Continuity of care by a primary midwife (caseload midwifery) increases women’s satisfaction with antenatal, intrapartum and postpartum care: results from the COSMOS randomised controlled trial. BMC Pregnancy Childbirth.

[19] Floris L, Irion O, Bonnet J, Mercier M-PP, De Labrusse C (2018). Comprehensive maternity support and shared care in Switzerland: comparison of levels of satisfaction. Women Birth.

[20] Fawsitt CG, Bourke J, Lutomski JE, Meaney S, McElroy B, Murphy R (2017). What women want: exploring pregnant women’s preferences for alternative models of maternity care. Health Policy.

[21] Moghasemi S, Vedadhir A, Simbar M (2018). Models for providing midwifery care and its challenges in the context of Iran. J Holistic Nurs Midwifery.

[22] Kaushik V, Walsh CA (2019). Pragmatism as a research paradigm and its implications for social work research. Soc Sci.

[23] Larkin P, Begley CM, Devane D (2012). ‘Not enough people to look after you’: an exploration of women's experiences of childbirth in the Republic of Ireland. Midwifery..

[24] Hsieh H-F, Shannon SE (2005). Three approaches to qualitative content analysis. Qual Health Res.

[25] Iran ministry of health and medical education. Integrated maternal health care. Department of population and family health. 7th ed; 2017.

[26] Graneheim UH, Lundman B (2004). Qualitative content analysis in nursing research: concepts, procedures and measures to achieve trustworthiness. Nurse Educ Today.

[27] Perriman N, Davis DL, Ferguson S (2018). What women value in the midwifery continuity of care model: a systematic review with meta-synthesis. Midwifery..

[28] Rawlett KE (2014). Journey from self-efficacy to empowerment. Health Care.

[29] Byrne J, Hauck Y, Fisher C, Bayes S, Schutze R (2014). Effectiveness of a mindfulness-based childbirth education pilot study on maternal self-efficacy and fear of childbirth. J Midwifery Women’s Health.

[30] Sigurðardóttir VL, Gamble J, Guðmundsdóttir B, Sveinsdóttir H, Gottfreðsdóttir H (2019). Processing birth experiences: a content analysis of women’s preferences. Midwifery..

[31] Aannestad M, Herstad M, Severinsson E. A meta-ethnographic synthesis of qualitative research on women's experience of midwifery care. Nurs Health Sci. 2020;22(2):171–83. 10.1111/nhs.12714. 10.1111/nhs.12714 32170804

[32] Peters M, Kolip P, Schäfers R (2020). A theory of the aims and objectives of midwifery practice: a theory synthesis. Midwifery..

[33] Moridi M, Pazandeh F, Hajian S, Potrata B (2020). Midwives’ perspectives of respectful maternity care during childbirth: a qualitative study. PLoS One.

[34] Iida M, Horiuchi S, Porter SE (2012). The relationship between women-centred care and women's birth experiences: a comparison between birth centres, clinics, and hospitals in Japan. Midwifery..

[35] Hunter A, Devane D, Houghton C, Grealish A, Tully A, Smith V (2017). Woman-centred care during pregnancy and birth in Ireland: thematic analysis of women’s and clinicians’ experiences. BMC Pregnancy Childbirth..

[36] Miller S, Abalos E, Chamillard M, Ciapponi A, Colaci D, Comandé D (2016). Beyond too little, too late and too much, too soon: a pathway towards evidence-based, respectful maternity care worldwide. Lancet.

[37] Hassan SJ, Sundby J, Husseini A, Bjertness E (2012). The paradox of vaginal examination practice during normal childbirth: Palestinian women’s feelings, opinions, knowledge and experiences. Reprod Health.

[38] Lyberg A, Severinsson E (2010). Fear of childbirth: mothers’ experiences of team-midwifery care–a follow-up study. J Nurs Manag.

[39] Iida M, Horiuchi S, Nagamori K. Women’s experience of receiving team-midwifery care in Japan: a qualitative descriptive study. Women Birth. 2020. 10.1016/j.wombi.2020.09.020. 10.1016/j.wombi.2020.09.020 33041236

[40] Skrondal TF, Bache-Gabrielsen T, Aune I. All that I need exists within me: a qualitative study of nulliparous Norwegian women's experiences with planned home birth. Midwifery. 2020;86:102705. 10.1016/j.midw.2020.102705. 10.1016/j.midw.2020.102705 32200282

[41] Karlström A, Nystedt A, Hildingsson I (2015). The meaning of a very positive birth experience: focus groups discussions with women. BMC Pregnancy Childbirth..

[42] Olza I, Leahy-Warren P, Benyamini Y, Kazmierczak M, Karlsdottir SI, Spyridou A (2018). Women’s psychological experiences of physiological childbirth: a meta-synthesis. BMJ Open.

