# Supplementary material for: Women’s perception of continuity of team midwifery care in Iran: a qualitative content analysis
Source: BMC Pregnancy Childbirth. 2021 Mar 2;21:173. doi: 10.1186/s12884-021-03666-z (PMC7922712; doi:10.1186/s12884-021-03666-z)
Supplement: Supplementary file 1 — Additional file 1. [file 12884_2021_3666_MOESM1_ESM.docx]

**Interview Guide**

**Introduction**

Thank you for accepting to participate in our interview. In this study, we want to ask your perception about the team midwifery care you have received during pregnancy, birth and postpartum. Your participation in the research is completely voluntary and you can cancel it in any part of the research. If you wish, the result of the research will be provided to you. We would like to audio record the interview to ensure that we do not miss any point. Your name and all information and statements are kept confidential and will not be shared with anyone. We want to audio record the interview, would that be OK?

**Personal Information:**

Name: __________ (optional)

Age: __________

Education level: __________

Employment status: __________

**Interview Questions:**

Would you describe your perception of care received from team midwives during pregnancy?

Would you describe your perception of care received from team midwives during childbirth?

Would you describe your perception of care received from team midwives during postpartum?
